# Rupture of an Ectopic Superior Mesenteric Vein Varix: A Case Report

**DOI:** 10.7759/cureus.87367

**Published:** 2025-07-06

**Authors:** Haruka Nishida, Yoshinori Matsuoka, Jumpei Fujimoto, Reiichi Ishikura, Koichi Ariyoshi

**Affiliations:** 1 Department of Emergency Medicine, International University of Health and Welfare Narita Hospital, Narita, JPN; 2 Department of Emergency Medicine, Kobe City Medical Center General Hospital, Kobe, JPN; 3 Department of Radiology, Kobe City Medical Center General Hospital, Kobe, JPN

**Keywords:** ectopic varices, interventional radiology, intraperitoneal hemorrhage, portal hypertension, superior mesenteric vein

## Abstract

Ectopic varices represent dilated portosystemic collaterals located outside the gastroesophageal region. Although typically asymptomatic and not requiring treatment, these varices can infrequently precipitate life-threatening intraperitoneal hemorrhage. We describe a case of intra-abdominal hemorrhage caused by rupture of a superior mesenteric vein (SMV) varix due to portal hypertension. An 84-year-old Japanese woman with end-stage hepatocellular carcinoma presented to our emergency department (ED) following a transient loss of consciousness. On arrival, her vital signs were stable despite a slightly distended abdomen. While awaiting diagnostic imaging, she suddenly went into shock. Repeat bedside ultrasonography revealed increased ascites compared with the initial evaluation, accompanied by progressive anemia and worsening metabolic acidosis. Resuscitation was initiated with type O packed red blood cell transfusion and resuscitative endovascular balloon occlusion of the aorta. She was then transferred to the computed tomography (CT) room in the ED, where resuscitative procedures were continued. Contrast-enhanced CT demonstrated massive hemorrhagic ascites and extravasation around the SMV. Although both interventional radiology and surgical intervention were considered, further invasive procedures were withheld given the patient's condition and her family's wishes. Cardiopulmonary arrest occurred two hours after the collapse. Management of intraperitoneal hemorrhage from ectopic varices in the ED remains a significant clinical challenge. Emergency physicians should consider this rare etiology in patients with prolonged portal hypertension. Interventional radiology, such as transjugular intrahepatic portosystemic shunt combined with embolization, may represent a viable treatment option.

## Introduction

Ectopic varices are defined as large portosystemic venous collaterals, excluding those in the gastroesophageal region [1-3]. Most are asymptomatic but may occasionally develop complications, including compression of surrounding organs, thrombosis of the varices, and gastrointestinal bleeding. Ruptures are rare among these complications, with a reported incidence of 2.2% [2]. However, once ectopic varices rupture, hemorrhage can be fatal due to excessive blood flow, with a mortality rate greater than 50% [3]. Optimal treatment depends on the location of the varicose veins, the local physician’s expertise, the cause, and the patient's condition. Therapeutic options include endoscopy, laparotomy, interventional radiology (IR), and medical therapy. Emergency laparotomy with the aim of ligating the bleeding varix, as well as transjugular intrahepatic portosystemic shunt (TIPS), has been reported as an effective management strategy for intraperitoneal rupture of an ectopic varix. We describe a rare case of intra-abdominal hemorrhage caused by rupture of a superior mesenteric vein (SMV) varix due to portal hypertension.

## Case presentation

An 84-year-old Japanese woman with hepatocellular carcinoma (HCC), receiving best supportive care, presented to our emergency department (ED) with transient loss of consciousness. She fainted shortly after standing up following dinner and was brought to the ED within one hour of symptom onset. She was independent in activities of daily living prior to presentation. According to her family, she had been diagnosed at her primary care hospital, where she had been regularly followed. A brief referral letter was urgently obtained from her primary care hospital at the time of presentation, indicating that she had terminal-stage hepatocellular carcinoma and liver cirrhosis of unknown etiology with portal hypertension. Her past medical history included hypertension, mild dementia, pulmonary emphysema, and interstitial pneumonia. On ED arrival, her vital signs were as follows: blood pressure 119/35 mmHg, heart rate 73 beats per minute, body temperature 36.2℃, oxygen saturation 99% on room air, respiratory rate 24 breaths per minute, and Glasgow Coma Scale score of 14 (E4V4M6), which was consistent with her baseline level of consciousness. She complained of mild discomfort in her abdomen. On physical examination, her abdomen was slightly distended but soft and not tender to palpation. Other than these findings, she had yellow eyelid conjunctiva and skin, and her extremities were cold. In addition, ultrasonography showed a small amount of ascites; however, no signs of abdominal aortic aneurysm, mass on the liver surface, or other remarkable findings in the abdominal organs were observed. Laboratory tests revealed the following: white blood cell count 8,600/μL, hemoglobin 7.8 g/dL, platelet count 171,000/μL, total bilirubin 6.3 mg/dL, aspartate aminotransferase 227 IU/L, alanine aminotransferase 304 IU/L, lactate dehydrogenase 351 IU/L, alkaline phosphatase 433 IU/L, hepatitis C virus antibody negative, hepatitis B surface antigen negative, prothrombin time INR 1.16, and activated partial thromboplastin time 29.3 seconds (Table 1).

**Table 1:** Laboratory data at the emergency department Laboratory data showed elevated hepatobiliary enzymes; however, there was no thrombocytopenia or evident coagulopathy. TP, total protein; Alb, albumin; T-Bil, total bilirubin; AST, aspartate aminotransferase; ALT, alanine aminotransferase; ALP, alkaline phosphatase; LDH, lactate dehydrogenase; BUN, blood urea nitrogen; Cr, creatinine; Na, sodium; K, potassium; Ca, calcium; Glu, glucose; CRP, C-reactive protein; WBC, white blood cell; RBC, red blood cell; Hb, hemoglobin; Hct, hematocrit; Plt, platelet; PT, prothrombin time; PT-INR, prothrombin time-international normalized ratio; APTT, activated partial thromboplastin time.

Variable	Value	Reference range
TP, g/dL	4.9	6.5-8.5
Alb, g/dL	2.4	3.9-4.9
T-Bil, mg/dL	6.3	0.2-1.2
AST, IU/L	227	8-40
ALT, IU/L	304	8-40
ALP, IU/L	433	100-340
LDH, IU/L	351	120-250
BUN, mg/dL	18.8	8.0-20.0
Cr, mg/dL	0.77	0.40-0.80
Na, mEq/L	143	136-148
K, mEq/L	3.8	3.5-5.3
Ca, mg/dL	8.2	8.0-10.0
Glu, mg/dL	151	70-110
WBC, ×10^3^/μl	8.6	3.9-9.8
RBC, ×10^4^/μl	211	350-510
Hb, g/dL	7.8	11.1-15.1
Hct, %	23.2	33.5-45.1
Plt, ×10^4^/μl	17.1	13-37
PT, %	78.2	80-125
PT-INR	1.16	
APTT, seconds	29.3	24.3-38.9
CRP, mg/dL	2.96	0.00-0.50

A venous blood gas analysis showed respiratory alkalosis and metabolic acidosis (pH 7.520, partial pressure of carbon dioxide 20.6 mmHg, bicarbonate 16.7 mmol/L, base excess −0.5 mmol/L, lactate 3.1 mmol/L (normal range, 0.5-1.6 mmol/L)).

**Figure 1:**
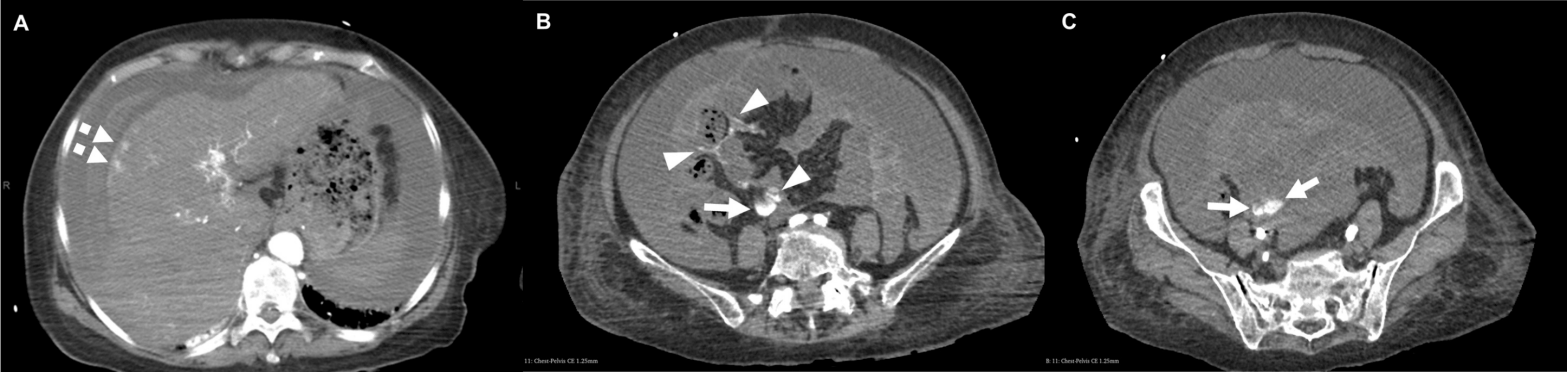
Abdominal contrast-enhanced CT scan showing intraperitoneal hemorrhage from ectopic varices rupture (A) A dense hematoma around the liver and parenchymal enhancements (dotted arrows), suggestive of infiltrative hepatocellular carcinoma. (B and C) An ectopic varix of the superior mesenteric vein (arrows) and contrast extravasation (arrowheads).

**Figure 2:**
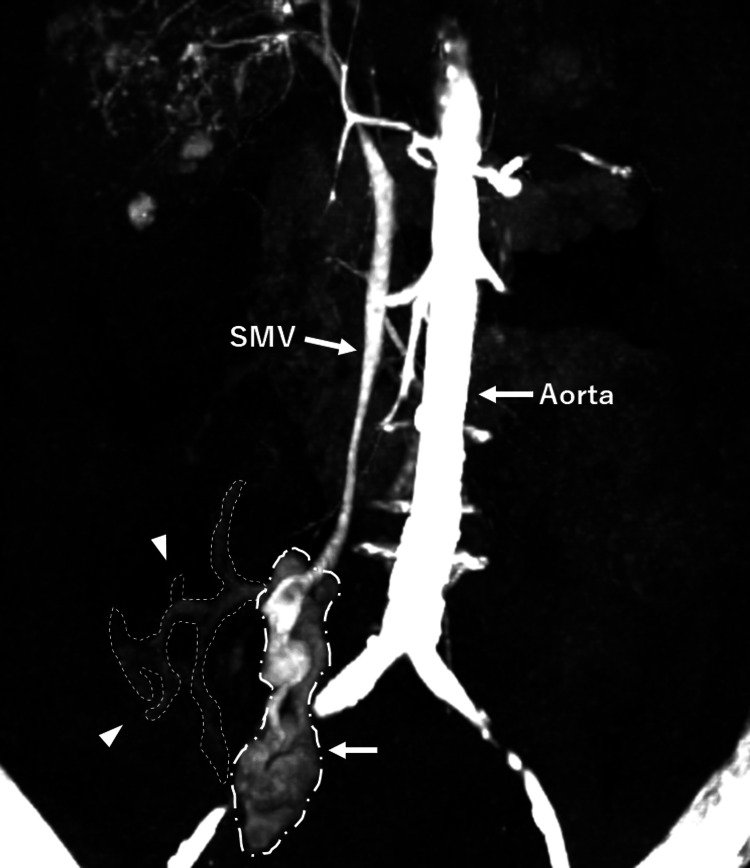
Three-dimensional reconstruction of abdominal CT scan showing rupture of the ectopic superior mesenteric vein varix This reconstructed CT image clearly reveals the superior mesenteric vein, the ruptured superior mesenteric vein (SMV) varices (arrows), and the associated extravasation and intraperitoneal hemorrhage (arrowheads).

Based on the above, we initially thought that we should rule out rupture of HCC as the cause of the patient’s transient loss of consciousness. While waiting for computed tomography (CT) in the ED, she suddenly lost consciousness after complaining of a bowel movement, and her blood pressure dropped to 77/65 mmHg with a heart rate of 50 beats per minute. Repeated bedside ultrasonography revealed an increased volume of ascites, while analysis of her blood gas showed progressive anemia and worsening metabolic acidosis (pH 7.235, partial pressure of carbon dioxide 29.0 mmHg, bicarbonate 11.8 mmol/L, hemoglobin 5.1 g/dL, base excess −14.2 mmol/L, lactate 10.7 mmol/L). We immediately started resuscitation with type O packed red blood cells. However, after 20 minutes of resuscitation following her collapse, her blood pressure dropped to 36/28 mmHg. Presuming rupture of HCC, we performed a resuscitative endovascular balloon occlusion of the aorta and took the patient to the CT room located inside the ED, where we continued resuscitative procedures. Simultaneously, we consulted gastroenterologists in the hospital. Contrast-enhanced CT did not reveal any findings of HCC rupture or abdominal aortic aneurysm rupture, but showed massive hemorrhagic ascites, extravasation around the SMV, an irregular liver surface, and splenomegaly (Figures 1, 2).

SMV varices were not identified at the time of initial interpretation by emergency physicians and gastroenterologists, and they were later confirmed by a radiologist. While we suspected that the bleeding site was on the periphery of the SMV, the patient had poor hepatic functional reserve and was hemodynamically unstable. We therefore discussed the situation with the gastroenterologists and determined that surgical treatment, such as laparotomy, was not indicated. We also considered IR procedures, including transjugular intrahepatic portosystemic shunt (TIPS) placement to reduce portal pressure and, if accessible, coil or n-butyl-2-cyanoacrylate embolization of the ruptured site. However, we discussed the patient’s condition with her family and reviewed her wishes, and then decided not to perform any further interventional treatment. The patient’s condition continued to deteriorate, and cardiopulmonary arrest occurred two hours after she collapsed in the ED.

## Discussion

The case presented here was an intra-abdominal hemorrhage due to rupture of SMV varices associated with portal hypertension. We highlight two important clinical learning points. First, when managing intra-abdominal hemorrhage in patients with prolonged portal hypertension, emergency physicians should consider this rare and catastrophic entity, namely, ectopic varices rupture, as a differential diagnosis. Second, although a definitive treatment strategy for this condition has not been established, IR procedures such as TIPS alone or TIPS in combination with embolization have been reported to be minimally invasive and effective [3-7], and may be positively considered for hemodynamically unstable patients with limited liver functional reserve.

The appropriate and timely diagnosis of rupture of ectopic varices is demanding due to its rarity and the difficulty in identifying the bleeding site in EDs. Ectopic varicose veins, defined as large portosystemic venous collaterals located anywhere other than the gastroesophageal region, occur infrequently [8,9]. They are often associated with prolonged portal hypertension, such as in liver cirrhosis [2,10], and can also result from obstruction of the mesenteric veins due to thrombosis or tumor invasion, or from conditions such as abdominal surgery, trauma, or pancreatitis [2,8]. Such a patient’s background should alert emergency physicians to include this rare but lethal entity in the differential diagnosis. Furthermore, it has been reported that identifying the bleeding site can be difficult, even when extravasation of contrast medium into the abdominal cavity on portal phase CT suggests intraperitoneal variceal hemorrhage. In some cases, laparotomy or autopsy has been required to diagnose the bleeding site with certainty [3,4]. In fact, the present case involved intra-abdominal hemorrhage caused by rupture of an SMV varix, but it was challenging for the emergency physicians and gastroenterologists to reach a definitive diagnosis while managing the patient in the ED.

Both surgical interventions and IR procedures may be considered as treatment strategies when resuscitating patients such as the present case. Laparotomy is a direct approach for managing intra-abdominal hemorrhage and has been the treatment of choice in many previous studies [2]. However, it is well known that such surgery carries high postoperative mortality, as these patients often have advanced-stage cirrhosis or are in a state of severe shock [3,11,12]. Patients are, therefore, sometimes not considered candidates for surgical intervention. In these cases, IR procedures should be considered as an alternative treatment. IR procedures have been reported to be effective and less invasive, and several approaches, including TIPS alone or TIPS combined with embolization, have been recommended in the literature [3-7]. Although TIPS alone has been shown to control bleeding in more than 90% of cases, rebleeding often occurs [5]. For this reason, previous reports have recommended that a combination of TIPS and antegrade variceal embolization is an optimal approach for managing hemorrhage from ectopic varices [3,5]. In the present case, unfortunately, we could not offer either surgical intervention or IR procedures, as the patient was in the terminal stage of HCC, in a state of severe shock, and her family did not wish her to undergo further invasive treatment.

## Conclusions

Emergency physicians should be mindful of the possibility of ectopic varices rupture as a source of intraperitoneal hemorrhage and should be especially vigilant in patients with chronic liver disease. The optimal management for such patients has yet to be established, as current knowledge is primarily derived from case reports and small case series. Therefore, each patient should be approached on a case-by-case basis, taking into account the patient’s condition, liver functional reserve, and the availability of experts and equipment at the institution. IR procedures, particularly TIPS combined with embolization, may be considered the first-line option for ruptured ectopic varicose veins in patients with advanced-stage liver disease.

## References

[1] Smith TJ, Morehouse DL (2011). Superior mesenteric vein aneurysm rupture. Vasc Endovascular Surg.

[2] Sfyroeras GS, Antoniou GA, Drakou AA, Karathanos C, Giannoukas AD (2009). Visceral venous aneurysms: clinical presentation, natural history and their management: a systematic review. Eur J Vasc Endovasc Surg.

[3] Watanabe M, Shibuya A, Kitamura Y (2008). Intraperitoneal bleeding due to rupture of the left gastric vein (LGV) in a patient with liver cirrhosis: a case report. Abdom Imaging.

[4] Olusola BF, McCashland TM, Seemayer TA, Sorrell MF (2002). Rectovesical ectopic varix intraperitoneal hemorrhage with fatal outcome. Am J Gastroenterol.

[5] Vangeli M, Patch D, Terreni N, Tibballs J, Watkinson A, Davies N, Burroughs AK (2004). Bleeding ectopic varices--treatment with transjugular intrahepatic porto-systemic shunt (TIPS) and embolisation. J Hepatol.

[6] Helmy A, Al Kahtani K, Al Fadda M (2008). Updates in the pathogenesis, diagnosis and management of ectopic varices. Hepatol Int.

[7] Vidal V, Joly L, Perreault P, Bouchard L, Lafortune M, Pomier-Layrargues G (2006). Usefulness of transjugular intrahepatic portosystemic shunt in the management of bleeding ectopic varices in cirrhotic patients. Cardiovasc Intervent Radiol.

[8] Starikov A, Bartolotta RJ (2015). Massive superior mesenteric venous aneurysm with portal venous thrombosis. Clin Imaging.

[9] Wolosker N, Zerati AE, Nishinari K, de Melo Galvão Filho M, Wolosker AM (2004). Aneurysm of superior mesenteric vein: case report with 5-year follow-up and review of the literature. J Vasc Surg.

[10] Akhter NM, Haskal ZJ (2012). Diagnosis and management of ectopic varices. Int J Gastrointest Interv.

[11] Kosowsky JM, Gibler WB (2000). Massive hemoperitoneum due to rupture of a retroperitoneal varix. J Emerg Med.

[12] Almadi MA, Almessabi A, Wong P, Ghali PM, Barkun A (2011). Ectopic varices. Gastrointest Endosc.

